# Elobixibat Improves Stool/Gas Distribution and Fecal Bile Acids in Older Adults With Chronic Constipation

**DOI:** 10.1002/jgh3.70223

**Published:** 2025-08-18

**Authors:** Noriaki Manabe, Emiko Bukeo, Takako Konishi, Maki Ayaki, Minoru Fujita, Ken Haruma

**Affiliations:** ^1^ Division of Endoscopy and Ultrasonography, Department of Laboratory Medicine Kawasaki Medical School Okayama Japan; ^2^ Department of General Internal Medicine 2 Kawasaki Medical School Okayama Japan

**Keywords:** bile acids, bowel stool and/or gas distribution, colonic motility and disorders, ileal bile acid transporter inhibitor, transabdominal ultrasonography

## Abstract

**Background:**

Bowel stool and/or gas distribution is an important indicator of bowel function. The mean colonic transverse diameter of the colon and rectum (constipation index) is a stool and/or gas distribution parameter assessed via transabdominal ultrasonography and an indirect indicator of colonic transit time. Elobixibat, an ileal bile acid transporter inhibitor, increases the number of bowel movements in chronic constipation (CC) patients.

**Aims:**

To determine whether the constipation index changes following treatment with elobixibat and evaluate the relationship between changes in the constipation index and fecal bile acid concentration.

**Methods:**

We conducted a prospective, randomized, double‐blind, placebo‐controlled trial in 17 CC patients aged ≥ 60 years who were administered elobixibat or placebo daily for 1 week. Changes in stool and/or gas distribution and stool bile acid concentrations were analyzed before and after treatment.

**Results:**

Elobixibat, but not placebo, significantly reduced the constipation index. A significant negative correlation was found between the change in the constipation index and the change in total fecal bile acid concentration.

**Conclusion:**

Elobixibat increases the total fecal bile acid concentration of CC patients, which results in shortened colonic transit time.

**Trial Registration:** JRCT ID: jRCTs061200030; https://rctportal.niph.go.jp/en/detail?trial_id=jRCTs061200030

**Abbreviations**
AC: ascending colon
BA: bile acid
CA: cholic acid
CC: chronic constipation
CI: constipation index
DC: descending colon
IBAT: ileal bile acid transporter
MRI: magnetic resonance imaging
R: rectum
S/G: stool and/or gas
SC: sigmoid colon
STC: slow transit constipation
TC: transverse colon
TGR5: G protein‐coupled receptor 5
TUS: transabdominal ultrasonography

## 1 Introduction

A key element of chronic constipation (CC) is the prolongation of colonic transit time associated with decreased colonic motility. CC is a common disease in older adults [1], and several recent reports have shown that CC is associated with a variety of diseases that occur frequently in older adults, such as frailty, cardiovascular disorders, and kidney disease [2, 3, 4]. Therefore, it is important to provide appropriate treatment to patients with CC to interrupt the vicious cycle involving the various diseases related to CC. At present, many physicians determine the optimal laxative for their patients via trial and error. However, because of the diversity of CC pathophysiology among patients, administering drugs that do not align with pathophysiology can cause side effects, such as abdominal pain, diarrhea, and ischemic enteritis [5]. This is because CC is a functional colorectal disease, and although treatment should ideally be tailored to each individual's condition.

Methods reported to date for determining the pathophysiology of CC include defecography, colonic transit time testing using radiopaque markers, and anorectal manometry, all of which have been validated to a certain degree [6]. However, such methods are not commonly used in the treatment of CC because of radiation exposure, equipment cost, and complexity of the procedure. Recent technological advances have led to the clinical application of transabdominal ultrasonography (TUS), a noninvasive, low‐cost, simple, and repeatable method, to diagnose and treat gastrointestinal diseases [7]. To date, there have been several reports on the clinical application of TUS in the treatment of CC [8, 9]. Previously, we reported that the mean transverse diameter of the colon and rectum (i.e., the constipation index [CI]), assessed using TUS, differs between healthy subjects and patients with CC and that the CI is an indirect indicator of colonic transit time [10].

A previous novel study using scintigraphy showed that patients with CC have a prolonged colonic transit time in the right‐sided colon [11]. Furthermore, a recent study using magnetic resonance imaging (MRI) showed that the volume of the ascending colon after administration of 1 L of Moviprep was significantly different between patients with functional constipation and those with constipation‐predominant irritable bowel syndrome, despite reporting the same constipation symptoms [12]. Moreover, patients with CC have been shown using MRI to have larger distal transverse and sigmoid colon diameters [13]. Therefore, evaluating stool and/or gas (S/G) distribution in the colon and rectum may serve as a new pathophysiological assessment for patients with CC.

Elobixibat, a bile acid (BA) transporter inhibitor, increases the amount of BA in the intestinal tract by inhibiting the ileal bile acid transporter (IBAT) at the terminal ileum, thereby partially inhibiting BA reabsorption, which improves not only the number of complete spontaneous bowel movements and stool consistency but also the quality of life of patients [14, 15]. However, changes in the S/G distribution in the colon and rectum following elobixibat administration and its relationship with the amount of change in fecal BAs remain unknown.

Therefore, we aimed to determine, in a double‐blind, placebo‐controlled trial, whether the CI (an indirect indicator of colonic transit time), as assessed by TUS, changes following treatment with elobixibat and evaluate the relationship between changes in the CI and fecal BA.

## 2 Materials and Methods

### 2.1 Ethics

The study conformed with the principles of the Declaration of Helsinki and the Ethics Guidelines for Clinical Research of the Ministry of Health, Labour and Welfare, Japan. The study was conducted as a post hoc analysis of a study approved by the Clinical Research Review Committee of Kawasaki Medical School (approval number: CRB6200004), separately approved by the Ethics Committee at Kawasaki Medical School (6199‐00), and written informed consent was obtained from all participants.

### 2.2 Study Design

We prospectively compared pre‐ and posttreatment CIs of older adult patients with CC aged ≥ 60 years who met the Rome IV criteria [16]. A prospective, randomized, parallel‐group, double‐blind, placebo‐controlled clinical trial was conducted at Kawasaki Medical School General Medical Center between October 2020 and May 2022.

### 2.3 Recruitment Method and Inclusion/Exclusion Criteria

The inclusion criteria for older adult patients with CC were: (i) diagnosis of functional constipation according to the Rome IV criteria and (ii) age ≥ 60 years. The exclusion criteria were: (i) patients with or suspected of having constipation due to any organic diseases or constipation with outlet obstructive constipation; (ii) suspected biliary obstruction or low level of bile secretion; (iii) inability to take bisacodyl suppository; (iv) malignancy; (v) history of hypersensitivity to elobixibat; and (vi) severe renal (creatinine > 2 mg/dL), hepatic (total bilirubin > 3 mg/dL, or aspartate aminotransferase or alanine aminotransferase > 100 U/L) or cardiac disease. A total of 17 patients were enrolled (Table 1).

**TABLE 1:** Baseline characteristics of enrolled participants.

	CC (*n* = 17)		*p*
	Elobixibat (*n* = 9)	Placebo (*n* = 8)	
Sex (M/F)	5/4	4/4	NS
Mean age (median, IQR)	69.0 (66.0–73.0)	67.0 (64.0–69.0)	NS
Height (cm) (median, IQR)	165.9 (159.7–168.3)	158.7 (151.6–166.4)	NS
Body mass (kg) (median, IQR)	65.1 (54.9–67.9)	49.9 (46.6–66.3)	NS
Preexisting condition	4 (44.4%)	4 (50.0%)	NS
Concomitant illness	4 (44.4%)	2 (25.0%)	
Duration of CC (years) (median, IQR)	30.0 (5.5–45.0)	8.0 (3.5–23.0)	
History of treatment for CC	3 (33.3%)	2 (25.0%)	NS
Number of spontaneous bowel movements per week (median, IQR)	2.80 (2.00–4.20)	3.50 (1.70–4.43)	NS
Number of complete spontaneous bowel movements per week (median, IQR)	0 (0.00–0.00)	0.58 (0.00–1.40)	NS
Bristol stool scale score (1/2/3/4/5/6/7)	3/0/3/1/2/0/0	1/4/1/2/0/0/0	NS

Abbreviations: CC, chronic constipation; IQR, interquartile range; M/F, male/female; NS, not significant.

### 2.4 Experimental Protocol

**FIGURE 1:**
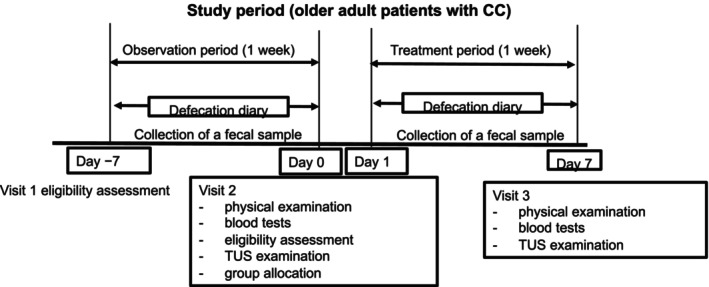
Experimental protocols. Protocol for patients with chronic constipation (CC). The defecation habits of the participants were recorded, blood tests were performed, and participants were temporarily enrolled at visit 1. After a 1‐week observation period, the participants were formally enrolled, and their symptoms were recorded at visit 2 (baseline). The participants underwent a physical examination and transabdominal ultrasonography (TUS) and were then randomly assigned to receive either placebo or elobixibat daily for 1 week. Patients underwent TUS after 1 week of treatment. Fecal bile acid concentrations were measured twice at any time during the observation period within 4–6 days of drug administration. During the 1‐week study period, participants were instructed to remain at home as usual (Days 1–5), stay at a hotel the day before TUS (Day 6), consume a designated meal (Day 6), and attend the TUS session on the final day (day 7, visit 3).

The defecation habits of the participants were recorded, blood tests were performed, and participants were provisionally enrolled at visit 1 (Figure 1). After a 1‐week observation period, participants were enrolled, and their symptoms were recorded at visit 2 (baseline). Participants underwent a physical examination and TUS before being randomly assigned to receive either a placebo or elobixibat daily for 1 week. Allocation was conducted by Satt Corporation, which is an entity not directly involved in the study, using a stratified substitution block method with sex as the allocation factor. For the placebo, we used a pill that was indistinguishable from the study drug.

TUS was performed again after 1 week of treatment. Fecal BA concentration (in a single random stool sample) was measured twice (at any time during the observation period and within 4–6 days of drug administration). If additional laxative therapy was deemed necessary, bisacodyl was administered as a single or double dose. During the 1‐week study period, participants were instructed to remain at home as usual (Days 1–5), to stay at a hotel 1 day before the test (Day 6), to consume a designated meal (Day 6), and to report for the TUS assessment on the final day (Day 7).

### 2.5 TUS Procedure

After an overnight fast, each subject was examined in a supine position the following morning. Subjects were instructed not to have a bowel movement until after the TUS examination. We measured the transverse diameter of four segments of the colon (ascending [AC], transverse [TC], descending [DC], and sigmoid colon [SC]) and the rectum (R) in each subject using TUS. TUS was performed using the Aplio i700 (Canon Medical Systems, Otawara, Japan) with 3.5 MHz curved array transducers. The distinction between colon and small intestine, as well as between AC and DC, is consistent with prior reports [10]. One physician independently performed the TUS, and assessors were blind to the results. The transverse diameters of each colonic segment were measured by TUS at three points (upper, middle and lower sites), and the average value was accepted as the transverse diameter of that segment. The S/G distribution was calculated according to the CI using the following equation, used previously [10]: CI = (AC + TC + DC + SC + *R*)/5.

### 2.6 Fecal BA Concentration

BA measurements were obtained by anonymously mailing a fecal sample to Techno Suruga Labs Inc. (Shizuoka, Japan). The total fecal BA concentration was defined as the sum of the individual BA concentrations, which were measured via liquid chromatography‐quadrupole time‐of‐flight mass spectrometry. Primary fecal BAs were defined as cholic acid (CA) + CDCA, and secondary fecal BAs were defined as deoxycholic acid + lithocholic acid. Fecal BA concentrations below the lower limit of quantification were imputed as missing values. Results are expressed as total BAs, conjugated moieties, and primary and secondary BAs per gram of feces.

### 2.7 Statistical Analysis

Continuous variables with a normal distribution are reported as means ± standard deviations, whereas those with a skewed distribution are reported as medians (interquartile ranges). For data with a normal distribution, Student's *t*‐test was used to compare the means of the two independent groups. Data that were not normally distributed were analyzed using the Mann–Whitney *U* test to compare the means of the two independent groups. To compare categorical data, we used the chi‐squared test with Yates's correction or Fisher's exact test. The Spearman test was used to examine correlations between changes in the CI and changes in total stool BA concentrations. In all analyses, *p* < 0.05 was considered statistically significant. All statistical analyses were performed using SPSS Version 17.0 (SPSS Inc., Chicago, IL).

## 3 Results

### 3.1 Participants

Seventeen participants were enrolled, of whom nine received elobixibat and eight received placebo (Table 1). No side effects were recorded in either group.

### 3.2 Differences in TUS Parameters Between the Two Groups Before and After Treatment

**FIGURE 2:**
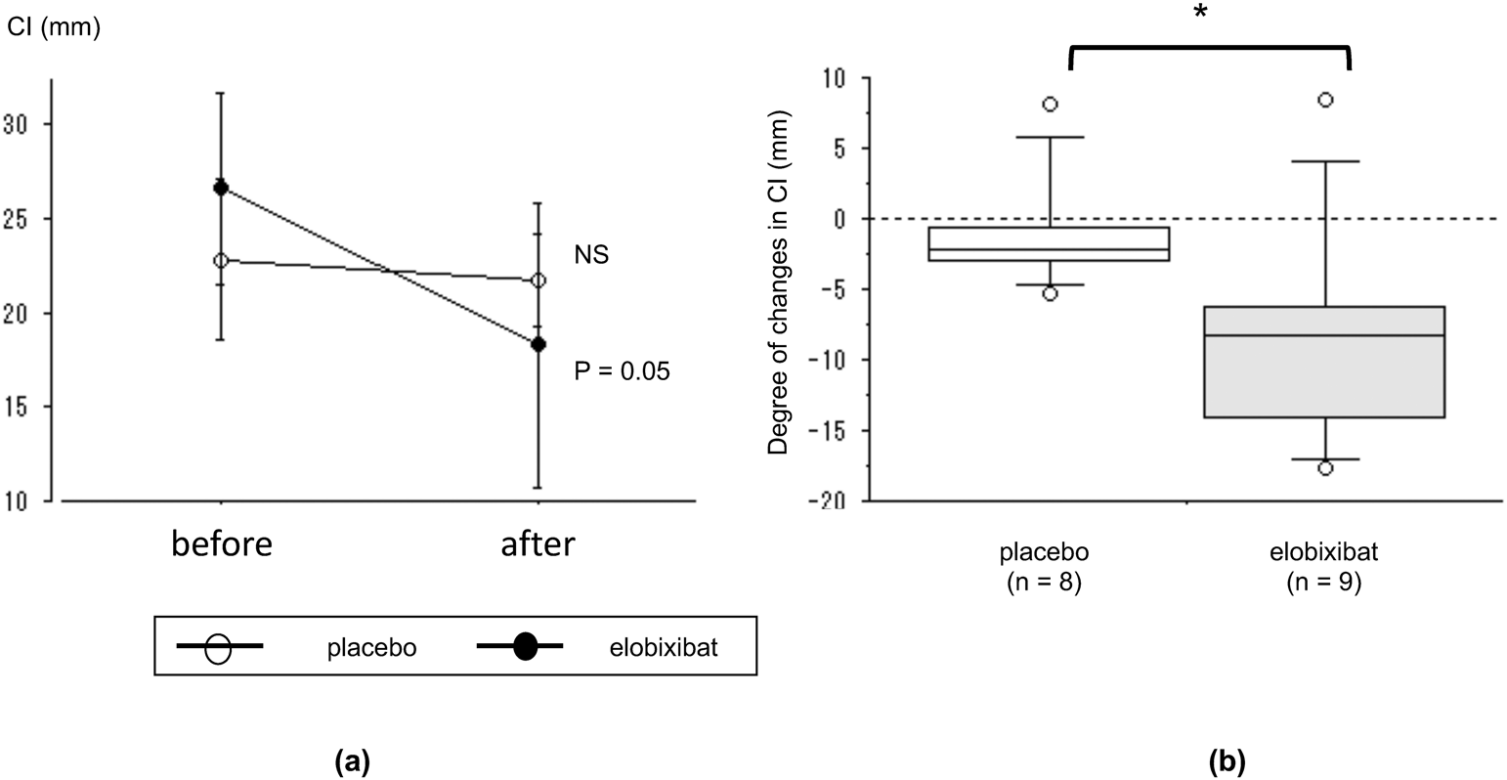
Change in the constipation index (CI) following treatment with placebo and elobixibat. (a) Time course of changes in CI. (b) Improvement of the CI. The therapeutic response in each group based on the CI was evaluated using Wilcoxon's signed‐rank test. *p < 0.05; **p < 0.01; NS, not significant.

There was no significant change in the CI after placebo treatment (*p* = 0.208), whereas the CI decreased significantly after elobixibat treatment (*p* = 0.05). The degree of change in the CI was also significantly different between the two groups (*p* = 0.018; Figure 2 and SI Table 1).

**FIGURE 3:**
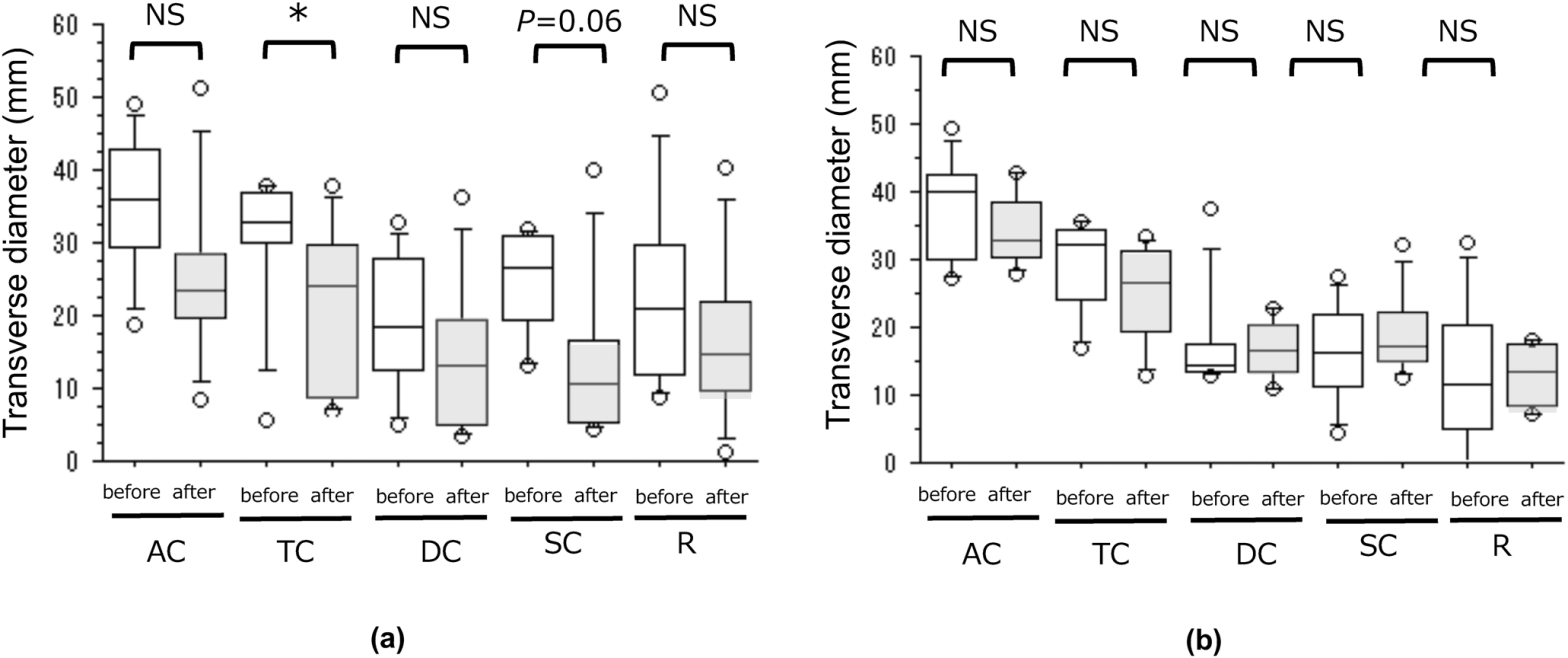
Changes in the stool and/or gas (S/G) distribution in each segment of the colon and rectum before and after 1 week of treatment with elobixibat (a) and placebo (b). AC, ascending colon; TC, transverse colon; DC, descending colon; SC, sigmoid colon; R, rectum; CC, chronic constipation. *p < 0.05; NS, not significant.

There was a significant difference in transverse diameters of the transverse colon before and after elobixibat treatment (*p* = 0.03), whereas there was a decreasing trend in transverse diameters of the sigmoid colon (*p* = 0.06). On the other hand, there were no significant differences in any of the sites before and after placebo treatment (Figure 3 and SI Table 2).

### 3.3 Differences in Total Stool BA Concentrations Between the Two Groups

**FIGURE 4:**
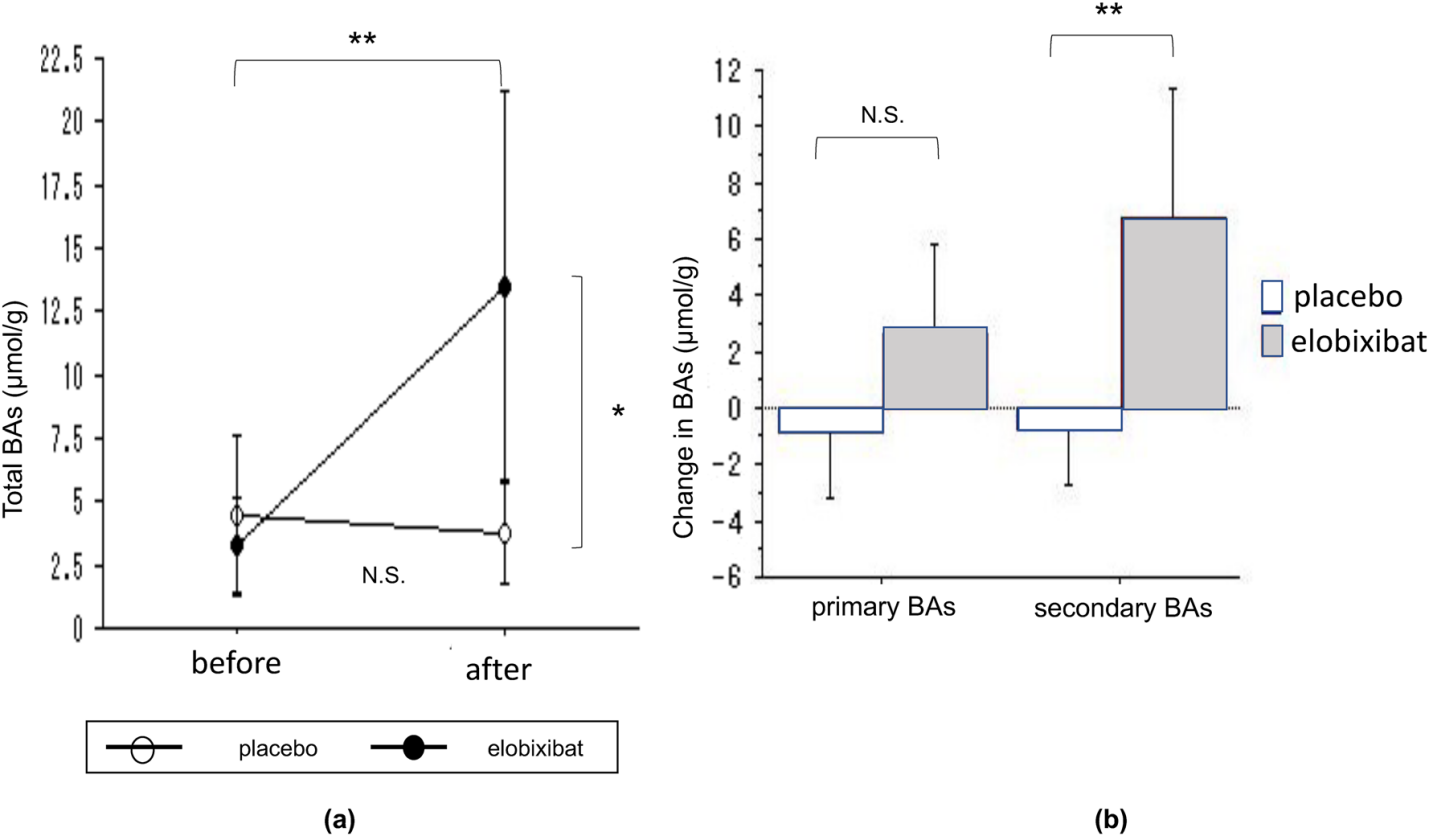
Differences in total stool bile acid (BA) concentrations between the two treatment groups. (a) Total fecal BA concentration before and after treatment. (b) Changes in primary and secondary BA concentrations after treatment in the two treatment groups. *p < 0.05; **p < 0.01; NS, not significant.

Total fecal BA concentrations of the elobixibat group increased significantly from baseline (*p* = 0.001), which was not observed in the placebo group. This increase was particularly pronounced for secondary BAs (primary BA, *p* = 0.06; secondary BA, *p* = 0.003) (Figure 4 and SI Table 3).

### 3.4 Relationship Between Changes in the CI and Changes in Total Stool BA Concentrations

**FIGURE 5:**
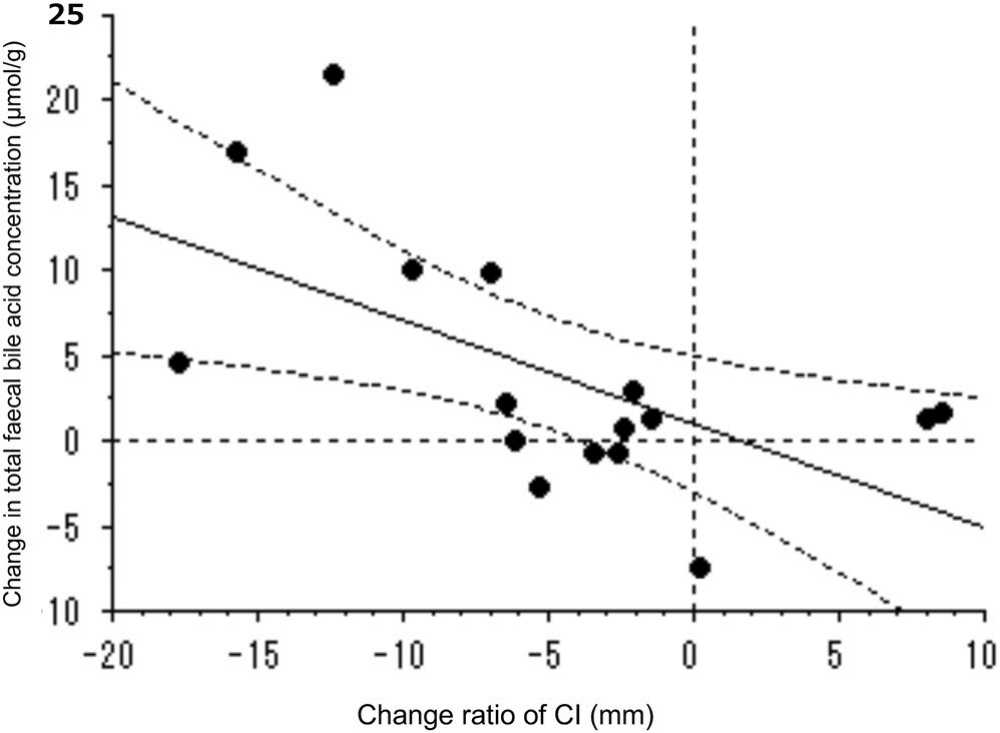
Relationship between the change in total stool bile acid (BA) concentrations and the change in the constipation index (CI) after treatment. There was a significant negative correlation between these two parameters.

Figure 5 shows the significant negative correlation between changes in the CI and changes in total stool BA concentrations (*r* = −0.564 [95% confidence interval: −0.828 to 0.094], *p* = 0.047).

## 4 Discussion

We showed that elobixibat, a BA transporter inhibitor, significantly reduced the mean transverse diameter of the colon and rectum by improving the S/G distribution of CC patients to a level similar to that of healthy subjects. Furthermore, this effect was significantly correlated with changes in total fecal BA concentration.

Given that the pathophysiology of CC is thought to be the result of altered colonic motility, abnormal S/G distribution is likely an important marker for the pathogenesis of CC. In clinical practice, the diagnosis and treatment of patients with CC with slow transit constipation (STC) are challenging. Previous studies have shown that scintigraphic patterns of colonic transit vary among patient groups. Krevsky et al. [17] and Stivland et al. [11] state that, in most patients, the delay in colonic transit time is in the right‐sided colon, whereas Roberts et al. [18] studied 37 patients with CC and showed that the delay was in the transverse colon and left flexure. Furthermore, other studies have reported delays in the left colon or distal part of the recto‐sigmoid colon, which is sometimes referred to as functional recto‐sigmoid colon obstruction [19]. Given such variability in the pathophysiology of STC, it is important to evaluate not only the total colonic transit time but also the transit time in each colonic segment.

Considering the above findings, it is clear that the evaluation of S/G distribution is crucial. We demonstrated that the administration of elobixibat improves both mean transverse colonic diameter—an indirect measure of colonic transit time—and S/G distribution in the colon and rectum, the latter of which reached a level comparable to that of healthy subjects. Our findings offer promise for pharmaceutical interventions based on S/G distribution in the colon and rectum.

Volumetric assessment of S/G distribution in the colon and rectum is a more precise method, and thus the optimal approach, because it would enable the identification of more substantial variation in S/G distribution between CC patients and healthy subjects. Recently, several studies have evaluated the pathophysiology of CC by measuring the transverse diameter of the colon and rectum using MRI; moreover, a relationship between symptoms and transverse diameter has been observed [13, 20]. In our study, we used a simpler, noninvasive evaluation method that is more suited to real‐world clinical settings. The transverse diameter of the colon and rectum, as evaluated by TUS, is reproducible and consistent with that evaluated by computed tomography [10]. Furthermore, the mean transverse diameter of the colon and rectum has been shown to correlate positively with colonic transit time, as measured by the marker method, using this technique [10]. Therefore, taken together, these findings suggest that transverse diameter measurement of the colon and rectum via TUS is a simple and repeatable pathological evaluation method for CC patients, a condition that is expected to be encountered increasingly frequently in daily practice [7].

When unabsorbed BAs enter the colon, they act on transmembrane G protein‐coupled receptor 5 (TGR5) on colonic epithelial cells. This activates the cystic fibrosis transmembrane conductance regulator via cyclic adenosine monophosphate and promotes the secretion of chloride ions into the intestinal tract. Additionally, 5‐hydroxytryptamine is released via TGR5 on enterochromaffin cells and acts on intrinsic sensory nerves in the submucosa and induces peristaltic reflexes. As a result, elobixibat induces both water secretion and peristalsis in the colon, leading to a defecatory effect [21, 22, 23, 24, 25]. Although a previous study demonstrated that elobixibat promotes colonic transit [26], our study is the first randomized controlled trial to evaluate changes in the S/G distribution in the colon and rectum. We also observed the colonic transit time‐promoting effect of elobixibat using the sonographic CI, an indirect measure of colonic transit time, and believe that this report is the first human clinical study to demonstrate an association between changes in the CI and fecal BA concentration.

Previous studies in patients with CC and healthy subjects treated with IBAT inhibitors have shown that the percentage of primary BAs (CA + CDCA) in the stool is higher after treatment than before [14, 27]. However, a recent study showed an increase in secondary BAs [28], which is consistent with our study. Possible reasons for this discrepancy include study design, patient demographics, and frequency of defecation during treatment [28].

For the stool samples for BA retrieved 4–6 days after treatment, bisacodyl can still be used as a single or double dose, which may have affected the BA and TUS results. In the current study, there was one patient each in the placebo and elobixibat groups who used bisacodyl once; moreover, there was no difference in bisacodyl use between the two groups. Therefore, it is unlikely that bisacodyl use affected our results.

This study has several limitations. First, we measured BA concentrations in random fecal samples rather than total stool BA excretions. Although uncertain, total BA excretion may correlate more strongly with the CI. Second, the TUS method does not take into account the water content of stools, which is considered for MRI‐based evaluations [29]. Although it is difficult to evaluate water content via TUS, there have been recent attempts to evaluate stool consistency (i.e., normal, hard, and no stools) using rectal ultrasound [30]. Although the amount of water in the stool cannot be quantified numerically, the above three‐level evaluation approach may be useful.

In conclusion, our findings suggested that elobixibat significantly improves S/G distribution in patients with CC, and this effect correlates with changes in total fecal BA concentration. In the future, the evaluation of S/G distribution will serve not only as an indicator of the objective efficacy of constipation medications but also as a basis for selection.

## Ethics Statement

The study conformed to the principles of the Declaration of Helsinki and the Ethics Guidelines for Clinical Research of the Ministry of Health, Labour and Welfare, Japan. The study was conducted as a post hoc analysis of a study approved by the Clinical Research Review Committee of Kawasaki Medical School (approval number: CRB6200004), separately approved by the Ethics Committee at Kawasaki Medical School (6199‐00), and written informed consent was obtained from all participants.

## Consent

Authorized.

## Conflicts of Interest

N.M. received honoraria from EA Pharma Co. Ltd. and Mochida Pharmaceutical Co. Ltd.

## Supporting information


**Supporting Information Table 1.** Change in the CI before and after treatment with placebo and elobixibat
**Supporting Information Table 2**. Changes in the S/G distribution in each segment of the colon and rectum before and after 1 week of treatment with elobixibat and placebo
**Supporting Information Table 3**. Fecal BA concentrations

## Data Availability

Data are held at Kawasaki Medical School, and the datasets used and/or analyzed during the current study will be made available by the corresponding author (NM) on reasonable request. Additional Supporting Information can be found online in the SI section.
