# Supplementary material for: Elobixibat Improves Stool/Gas Distribution and Fecal Bile Acids in Older Adults With Chronic Constipation
Source: JGH Open. 2025 Aug 18;9(8):e70223. doi: 10.1002/jgh3.70223 (PMC12358735; doi:10.1002/jgh3.70223)
Supplement: Supplementary file 1 — Supporting Information Table 1. Change in the CI before and after treatment with placebo and elobixibat Supporting Information Table 2. Changes in the S/G distribution in each segment of the colon and rectum before and after 1 week of treatment with elobixibat and placebo Supporting Information Table 3. Fecal BA concentrations [file JGH3-9-e70223-s001.docx]

**Supplementary Table 1. Change in the CI before and after treatment with placebo and elobixibat**

|  |
| --- |

|  |
| --- |

|  | Elobixibat (n=9) | | | | Placebo (n=8) | | | | diff | 95%CI | *P*-value |
| --- | --- | --- | --- | --- | --- | --- | --- | --- | --- | --- | --- |
|  | mean | SD | median | IQR | mean | SD | median | IQR |  |  |  |
| CI value after treatment | 17.1 | 7.4 | 15.0 | 12.7-18.9 | 21.9 | 2.5 | 22.6 | 20.7-23.6 | -4.81 | -10.7—2.1 | 0.100 |
| Amount of changes in CI | -9.7 | 8.2 | -10.3 | -15.2—6.02 | -0.9 | 3.7 | -1.6 | -3.1—0.3 | -8.82 | -15.5—2.1 | 0.013 |

CI, constipation index; SD, standard deviation; IQR, interquartile range; 95%CI, 95% confidence interval; diff, difference

**Supplementary Table 2. Changes in the S/G distribution in each segment of the colon and rectum before and after 1 week of treatment with elobixibat and placebo**

| elobixibat | | n | mean | sd | median | IQR | *P*-value |
| --- | --- | --- | --- | --- | --- | --- | --- |
| AC (mm) | baseline | 9 | 36.3 | 8.6 | 36.5 | 30.5-43.5 | 0.13 |
|  | after Tx | 9 | 23.8 | 12.3 | 23.7 | 14.6-27.3 |  |
| TC (mm) | baseline | 9 | 30.2 | 9.1 | 33.9 | 28.0-35.4 | 0.02 |
|  | after Tx | 9 | 19.5 | 12.5 | 21.1 | 7.1-27.9 |  |
| DC (mm) | baseline | 9 | 18.5 | 8.5 | 17.3 | 13.5-19.1 | 0.24 |
|  | after Tx | 9 | 13.7 | 10.9 | 13.0 | 4.9-18.0 |  |
| SC (mm) | baseline | 9 | 21.7 | 9.3 | 23.5 | 14.7-30.6 | 0.06 |
|  | after Tx | 9 | 13.0 | 11.1 | 9.2 | 5.6-12.8 |  |
| R (mm) | baseline | 9 | 17.8 | 126 | 14.2 | 10.3-24.8 | 0.41 |
|  | after Tx | 9 | 16.0 | 11.3 | 14.2 | 11.5-17.8 |  |
| placebo | | n | mean | sd | median | IQR | *P*-value |
| AC (mm) | baseline | 9 | 40.1 | 5.6 | 38.5 | 30.1-42.5 | 0.28 |
|  | after Tx | 9 | 33.7 | 5.11 | 32.8 | 30.9-36.6 |  |
| TC (mm) | baseline | 9 | 33.2 | 5.1 | 33.9 | 25.0-35.2 | 0.33 |
|  | after Tx | 9 | 25.2 | 6.86 | 26.2 | 19.4-31.8 |  |
| DC (mm) | baseline | 9 | 16.5 | 7.7 | 17.1 | 13.5-19.1 | 0.86 |
|  | after Tx | 9 | 17.4 | 4.72 | 17.6 | 14.2-20.8 |  |
| SC (mm) | baseline | 9 | 13.6 | 9.3 | 15.2 | 13.5-22.6 | 0.32 |
|  | after Tx | 9 | 18.8 | 6.69 | 16.7 | 14.1-21.3 |  |
| R (mm) | baseline | 9 | 8.3 | 5.5 | 7.4 | 5.23-20.1 | 0.95 |
|  | after Tx | 9 | 13.0 | 4.92 | 13.4 | 8.25-17.25 |  |

CC, chronic constipation; Tx, treatment with elobixibat; SD, standard deviation; IQR, interquartile range; AC, ascending colon; TC, transverse colon; DC, descending colon; SC, sigmoid colon; R, rectum.

**Supplementary Table 3. Faecal BA concentrations**

|  |  |  | N | mean | SD | median | IQR | difference | 95% CI | *P*-value |
| --- | --- | --- | --- | --- | --- | --- | --- | --- | --- | --- |
| CA (μmol/g) | Observation period | elobixibat | 8 | 0.000 | 0.000 | 0.000 | 0.000 – 0.000 | −0.961 | −2.9474 – 1.0249 | 0.3169 |
|  |  | placebo | 8 | 0.961 | 2.619 | 0.000 | 0.000 – 0.125 |  |  |  |
|  | Treatment period | elobixibat | 9 | 1.790 | 2.910 | 0.390 | 0.000 – 1.880 | 1.414 | −0.8213 – 3.6488 | 0.1976 |
|  |  | placebo | 8 | 0.376 | 0.546 | 0.000 | 0.000 – 0.820 |  |  |  |
|  | Difference | elobixibat | 8 | 1.011 | 1.856 | 0.225 | 0.000 – 1.155 | 1.596 | −0.7616 – 3.9541 | 0.1685 |
|  |  | placebo | 8 | −0.585 | 2.495 | 0.000 | 0.000 – 0.295 |  |  |  |
| CDCA (μmol/g) | Observation period | elobixibat | 8 | 0.000 | 0.000 | 0.000 | 0.000 – 0.000 | −0.404 | −1.2395 – 0.4320 | 0.3177 |
|  |  | placebo | 8 | 0.404 | 1.102 | 0.000 | 0.000 – 0.050 |  |  |  |
|  | Treatment period | elobixibat | 9 | 1.262 | 1.938 | 0.110 | 0.000 – 1.860 | 0.940 | −0.6098 – 2.4892 | 0.2157 |
|  |  | placebo | 8 | 0.323 | 0.710 | 0.000 | 0.000 – 0.270 |  |  |  |
|  | Difference | elobixibat | 8 | 0.790 | 1.414 | 0.080 | 0.000 – 1.120 | 0.871 | −0.5730 – 2.3155 | 0.2166 |
|  |  | placebo | 8 | −0.081 | 1.276 | 0.000 | 0.000 – 0.005 |  |  |  |
| DCA (μmol/g) | Observation period | elobixibat | 8 | 1.423 | 0.991 | 1.135 | 0.955 – 1.620 | −0.228 | −1.513 – 1.0584 | 0.7100 |
|  |  | placebo | 8 | 1.650 | 1.376 | 1.380 | 0.610 – 2.580 |  |  |  |
|  | Treatment period | elobixibat | 9 | 7.318 | 4.557 | 7.850 | 3.150 – 9.980 | 5.927 | 2.3924 – 9.4607 | 0.0028 |
|  |  | placebo | 8 | 1.391 | 1.102 | 1.255 | 0.435 – 2.385 |  |  |  |
|  | Difference | elobixibat | 8 | 5.563 | 4.565 | 5.115 | 1.985 – 8.145 | 5.821 | 2.2060 – 9.4365 | 0.0039 |
|  |  | placebo | 8 | −0.259 | 1.376 | −0.100 | −0.885 – 0.640 |  |  |  |
| UDCA (μmol/g) | Observation period | elobixibat | 8 | 0.000 | 0.000 | 0.000 | 0.000 – 0.000 | −0.134 | −0.394 – 0.1266 | 0.2892 |
|  |  | placebo | 8 | 0.134 | 0.343 | 0.000 | 0.000 – 0.045 |  |  |  |
|  | Treatment period | elobixibat | 9 | 0.861 | 1.322 | 0.000 | 0.000 – 1.090 | 0.684 | −0.3493 – 1.7165 | 0.1788 |
|  |  | placebo | 8 | 0.178 | 0.367 | 0.000 | 0.000 – 0.180 |  |  |  |
|  | Difference | elobixibat | 8 | 0.509 | 0.848 | 0.000 | 0.000 – 0.860 | 0.465 | −0.1814 – 1.1114 | 0.1452 |
|  |  | placebo | 8 | 0.044 | 0.084 | 0.000 | 0.000 – 0.055 |  |  |  |
| HDCA (μmol/g) | Observation period | elobixibat | 8 | 0.0 | 0.0 | 0.0 | 0.0 – 0.0 | 0.0 | 0.0 – 0.0 | - |
|  |  | placebo | 8 | 0.0 | 0.0 | 0.0 | 0.0 – 0.0 |  |  |  |
|  | Treatment period | elobixibat | 9 | 0.0 | 0.0 | 0.0 | 0.0 – 0.0 | 0.0 | 0.0 – 0.0 | - |
|  |  | placebo | 8 | 0.0 | 0.0 | 0.0 | 0.0 – 0.0 |  |  |  |
|  | Difference | elobixibat | 8 | 0.0 | 0.0 | 0.0 | 0.0 – 0.0 | 0.0 | 0.0 – 0.0 | - |
|  |  | placebo | 8 | 0.0 | 0.0 | 0.0 | 0.0 – 0.0 |  |  |  |
| LCA (μmol/g) | Observation period | elobixibat | 8 | 2.005 | 1.638 | 1.420 | 1.065 – 2.165 | 0.728 | −0.7412 – 2.1962 | 0.3060 |
|  |  | placebo | 8 | 1.278 | 1.330 | 1.330 | 0.335 – 2.045 |  |  |  |
|  | Treatment period | elobixibat | 9 | 2.202 | 2.410 | 2.410 | 1.270 – 2.630 | 0.811 | −0.4680 – 2.0899 | 0.1966 |
|  |  | placebo | 8 | 1.391 | 0.910 | 0.910 | 0.335 – 2.610 |  |  |  |
|  | Difference | elobixibat | 8 | 0.031 | 0.105 | 0.105 | −0.290 – 0.870 | −0.083 | −1.5831 – 1.4181 | 0.9078 |
|  |  | placebo | 8 | 0.114 | 0.095 | 0.095 | −0.470 – 0.690 |  |  |  |
| DA (μmol/g) | Observation period | elobixibat | 8 | 0.0 | 0.0 | 0.0 | 0.0 – 0.0 | 0.0 | 0.0 – 0.0 | - |
|  |  | placebo | 8 | 0.0 | 0.0 | 0.0 | 0.0 – 0.0 |  |  |  |
|  | Treatment period | elobixibat | 9 | 0.0 | 0.0 | 0.0 | 0.0 – 0.0 | 0.0 | 0.0 – 0.0 | - |
|  |  | placebo | 8 | 0.0 | 0.0 | 0.0 | 0.0 – 0.0 |  |  |  |
|  | Difference | elobixibat | 8 | 0.0 | 0.0 | 0.0 | 0.0 – 0.0 | 0.0 | 0.0 – 0.0 | - |
|  |  | placebo | 8 | 0.0 | 0.0 | 0.0 | 0.0 – 0.0 |  |  |  |
| isoUDCA (μmol/g) | Observation period | elobixibat | 8 | 0.0 | 0.0 | 0.0 | 0.0 – 0.0 | 0.0 | 0.0 – 0.0 | - |
|  |  | placebo | 8 | 0.0 | 0.0 | 0.0 | 0.0 – 0.0 |  |  |  |
|  | Treatment period | elobixibat | 9 | 0.0 | 0.0 | 0.0 | 0.0 – 0.0 | 0.0 | 0.0 – 0.0 | - |
|  |  | placebo | 8 | 0.0 | 0.0 | 0.0 | 0.0 – 0.0 |  |  |  |
|  | Difference | elobixibat | 8 | 0.0 | 0.0 | 0.0 | 0.0 – 0.0 | 0.0 | 0.0 – 0.0 | - |
|  |  | placebo | 8 | 0.0 | 0.0 | 0.0 | 0.0 – 0.0 |  |  |  |
| Total BAs (μmol/g) | Observation period | elobixibat | 8 | 3.428 | 2.536 | 2.360 | 2.225 – 3.350 | −0999 | −4.4137 – 2.4162 | 0.5406 |
|  |  | placebo | 8 | 4.426 | 3.722 | 4.120 | 1.610 – 5.645 |  |  |  |
|  | Treatment period | elobixibat | 9 | 13.433 | 9.839 | 11.880 | 4.090 – 19.530 | 9.775 | 2.1275 – 17.2416 | 0.0157 |
|  |  | placebo | 8 | 3.659 | 2.486 | 3.940 | 1.160 – 6.100 |  |  |  |
|  | Difference | elobixibat | 8 | 7.904 | 7.901 | 5.945 | 1.695 – 13.330 | 8.671 | 2.2249 – 15.1176 | 0.0120 |
|  |  | placebo | 8 | −0.768 | 3.137 | −0.055 | −1.710 – 0.925 |  |  |  |
| primary BAs (μmol/g) | Observation period | elobixibat | 8 | 0.000 | 0.000 | 0.000 | 0.000 – 0.000 | −1.365 | −4.1869 – 1.4569 | 0.3171 |
|  |  | placebo | 8 | 1.365 | 3.721 | 0.000 | 0.000 – 0.175 |  |  |  |
|  | Treatment period | elobixibat | 9 | 3.052 | 4.837 | 0.540 | 0.000 – 3.740 | 2.353 | −1.4028 – 6.1097 | 0.2016 |
|  |  | placebo | 8 | 0.699 | 1.203 | 0.000 | 0.000 – 1.090 |  |  |  |
|  | Difference | elobixibat | 8 | 1.801 | 3.262 | 0.325 | 0.000 – 2.255 | 2.468 | −1.2712 – 6.2062 | 0.1788 |
|  |  | placebo | 8 | −0.666 | 3.697 | 0.000 | 0.000 – 0.300 |  |  |  |
| secondary BAs (μmol/g) | Observation period | elobixibat | 8 | 3.428 | 2.536 | 2.360 | 2.225 – 3.350 | 0.500 | −2.0805 – 3.0805 | 0.6840 |
|  |  | placebo | 8 | 2.928 | 2.269 | 2.710 | 0.945 – 5.195 |  |  |  |
|  | Treatment period | elobixibat | 9 | 9.520 | 5.591 | 10.480 | 4.090 – 13.510 | 6.738 | 2.1931 – 11.2819 | 0.0065 |
|  |  | placebo | 8 | 2.783 | 2.353 | 2.525 | 0.885 – 4.545 |  |  |  |
|  | Difference | elobixibat | 8 | 5.594 | 5.530 | 3.475 | 1.695 – 9.015 | 5.739 | 1.2522 – 10.2253 | 0.0158 |
|  |  | placebo | 8 | −0.145 | 2.103 | −0.350 | −1.710 – 1.545 |  |  |  |

N, number; SD, standard deviation; IQR, interquartile range; CI, confidence interval; CA, cholic acid; CDCA, chenodeoxycholic acid; DCA, deoxycholic acid; UDCA, ursodeoxycholic acid; HDCA**,** hyodeoxycholic acid; LCA, lithocholic acid; DA, dehydrocholic acid: BAs, bile acids. Primary BAs = CA + CDCA; secondary BAs = DCA + LCA + UDCA.
